# ResFinder – an open online resource for identification of antimicrobial resistance genes in next-generation sequencing data and prediction of phenotypes from genotypes

**DOI:** 10.1099/mgen.0.000748

**Published:** 2022-01-24

**Authors:** Alfred Ferrer Florensa, Rolf Sommer Kaas, Philip Thomas Lanken Conradsen Clausen, Derya Aytan-Aktug, Frank M. Aarestrup

**Affiliations:** ^1^​ National Food Institute, Technical University of Denmark, DK-2800 kgs. Lyngby, Denmark

**Keywords:** antimicrobial resistance genes, bioinformatics, online tool

## Abstract

Antimicrobial resistance (AMR) is one of the most important health threats globally. The ability to accurately identify resistant bacterial isolates and the individual antimicrobial resistance genes (ARGs) is essential for understanding the evolution and emergence of AMR and to provide appropriate treatment. The rapid developments in next-generation sequencing technologies have made this technology available to researchers and microbiologists at routine laboratories around the world. However, tools available for those with limited experience with bioinformatics are lacking, especially to enable researchers and microbiologists in low- and middle-income countries (LMICs) to perform their own studies. The CGE-tools (Center for Genomic Epidemiology) including ResFinder (https://cge.cbs.dtu.dk/services/ResFinder/) was developed to provide freely available easy to use online bioinformatic tools allowing inexperienced researchers and microbiologists to perform simple bioinformatic analyses. The main purpose was and is to provide these solutions for people involved in frontline diagnosis especially in LMICs. Since its original publication in 2012, ResFinder has undergone a number of improvements including improvement of the code and databases, inclusion of point mutations for selected bacterial species and predictions of phenotypes also for selected species. As of 28 September 2021, 820 803 analyses have been performed using ResFinder from 61 776 IP-addresses in 171 countries. ResFinder clearly fulfills a need for several people around the globe and we hope to be able to continue to provide this service free of charge in the future. We also hope and expect to provide further improvements including phenotypic predictions for additional bacterial species.

## Data Summary

ResFinder web-service: https://cge.food.dtu.dk/services/ResFinder/.

ResFinder software repository: https://bitbucket.org/genomicepidemiology/resfinder.git.

ResFinder database repository: https://bitbucket.org/genomicepidemiology/resfinder_db.git.

PointFinder database repository: https://bitbucket.org/genomicepidemiology/pointfinder_db.git.

CGEcore repository: https://bitbucket.org/genomicepidemiology/cge_core_module.git.

KMA repository: https://bitbucket.org/genomicepidemiology/kma.git.

blast: https://ftp.ncbi.nlm.nih.gov/blast/executables/blast+/.

**Significance as a BioResource to the community**

Reliable and easy identification of antimicrobial resistance genes (ARGs) in next-generation sequencing (NGS) data is important for surveillance and research into antimicrobial resistance. Here we present ResFinder, an online service allowing users with limited bioinformatics experience to identify ARGs in NGS-data hereby expanding the scientific and geographical community with access to such analyses. ResFinder consists of a manually curated database of ARGs and a bioinformatics pipeline, both of which can be downloaded and used independently. ResFinder is under continuous development and recent features include predictions of phenotypes based on the identified ARGs, which are expected to be useful for those performing surveillance and perhaps in the future to direct treatment. The ResFinder software and database is freely available and open source.

## Introduction

Antimicrobial resistance (AMR) is one of the greatest threats against human and animal health [1–4]. The ability to reliably identify resistance in bacterial isolates is essential for appropriate treatment as well as surveillance [1].

Antimicrobial susceptibility testing has, since originally being developed by Alexander Fleming, mainly been done using phenotypic testing using disc diffusion or determinations of the MIC by growth on plates, or in broth at different concentrations. Large efforts have gone into optimizing and standardizing phenotypic testing. The methodology has proven very valuable over time despite the poor reproducibility [5–7]. In particular, for less experienced or laboratories under establishment, e.g. in the low- and middle-income countries (LMICs), quality control and standardization of the phenotypic testing can be a challenge [5–7].

For surveillance purposes, the identification of the specific genes encoding AMR have also been valuable in understanding the local and global emergence and transmission of AMR [8, 9].

The rapid development in next-generation sequencing (NGS) enables laboratories, clinicians and researchers to obtain the complete sequence of bacterial isolates or even entire microbiomes with affordable prices and short turn-around times. This could potentially provide access to knowledge about all antimicrobial resistance genes (ARGs), which would be very valuable for surveillance and epidemiology. In addition, several studies have suggested that prediction of susceptibility profiles based on identification of the ARGs has a very high concordance with phenotypic testing [10–16]. Particularly in LMICs, where phenotypic testing is not yet standardized, NGS-based methods might be a major benefit. However, limited resources for the bioinformatic analyses is a major obstacle for these countries.

The CGE-tools [17–21], including ResFinder [22, 23], was originally developed to provide web-based, open and easily accessible bioinformatic analyses especially for less experienced scientists and frontline laboratories particularly in LMICs. In the following, we describe the purpose, early development of ResFinder, changes and improvement over time, as well as the current and future directions, also in the context of the multiple novel bioinformatic solutions and databases that have or are being created.

### Background, intention and original target groups

The Technical University of Denmark has since 2000 functioned as a WHO Collaborating Centre for Antimicrobial Resistance. We have through numerous training courses and collaborations with laboratories and individuals in particular LMICs observed the challenges in performing standardized susceptibility testing of clinical pathogens [5–7]. We have also observed the difficulties in sharing data [24, 25], especially when conventional results are stored locally in different paper formats using national languages.

Around a decade ago, the rapid developments in next-generation sequencing (NGS) had made this technology so comparatively cheap that it was likely that it could within a few years be available not only in research laboratories, but also in the veterinary and human frontline diagnostic including LMICs [26, 27]. This provides several potential advantages, including the following:

- Implementation of a common data standard that can easily be shared electronically.
- Creation of a common language avoiding biases from individual human interpretations.
- One analysis platform that could generate the entire information for an isolate, avoiding the need to invest in separate equipment for identification, susceptibility testing and typing.

However, while the technology might become available, it seemed unrealistic that a sufficient number of experienced bioinformaticians could be educated to create a critical mass at each and every laboratory, including the clinical frontline across the globe. However, a potential solution to mitigate this would be if it was possible to create simple and easy to use web-based bioinformatic solutions freely accessible for the frontline in LMICs. The Center for Genomic Epidemiology (CGE) was created in an attempt to develop such open-source solutions.

Thus the purpose of the CGE-tools was to create simple and easy to use bioinformatic analyses that were as follows:

- Open source and available free of charge.
- Targeting scientist and frontline diagnostic with no or limited bioinformatic experience in LMICs.
- Providing simple upload and output features.
- Contributing to capacity building especially in LMICs.
- Contributing to global standardization and data sharing.

## Development

### Initial development

When ‘databases’ are referred to in this review, it is not used as in traditional information technology terms. Here a database refers to a structured collection of related data. The ResFinder databases consist of csv, tsv, and FASTA formatted raw text files (see description below).

The CGE-tools consists of a pipeline and different databases with known genes. Originally, we intended to use the CARD-database [28] also for the original ResFinder. However, at that time it was uncertain whether CARD would be continuously updated and CARD in some cases was too inclusive with many genes that might be important to detect for research purposes, but were not or less clinically relevant. Thus, using CARD as a basis we decided to also create our own database for ResFinder. Based on literature searches we manually curated the database, with the attempt to only include ARGs that had been horizontally acquired [22].

The first versions of ResFinder were written in Perl and used blast [29] for aligning input sequences against the ResFinder database of ARGs [23]. The first versions were written and maintained by different PhD students and PostDocs. This is common practice in a research group, but it resulted in a fragmented and hard to maintain code.

### Making ResFinder more maintainable

As the popularity and more importantly the need for ResFinder rose, the need for an improved code also rose, as it was hard to debug and update the older versions of the code. In version 3 of ResFinder, the entire code was rewritten in Python and cleaned up in the process. However, this is an ongoing process and ResFinder is still undergoing code revisions in order to make it more maintainable and stable.

### Inclusion of selected point mutations

AMR can be acquired either through horizontal gene transfer or as mutations in already existing genes. In contrast to horizontally transmitted ARGs, identifying mutations leading to AMR requires species-specific databases. With the purpose of improving surveillance and detection of AMR in foodborne pathogens, we developed PointFinder that identified selected antimicrobial resistance encoding mutations in *Salmonella *, *Escherichia coli* and *Campylobacter jejuni* and *Campylobacter coli* [30]. This was initially a service separate from ResFinder, but has now been included.

### Gene and mutation finding by aligning raw read data

In older versions of ResFinder, only assembled genomes in FASTA format were accepted as input. Users of the web-service were also able to upload raw data. However, it was assembled with Velvet [31] and later Spades [32] prior to alignment with blast [29]. Assembling read data is very resource demanding compared to mapping and aligning data, especially when only ARGs, composing <1% of the reads, are targeted. In general, *de novo* assembly requires more read data to produce high-quality assemblies, while being prone to errors with the additional assembly step. In order for ResFinder to analyse raw read data directly, a new alignment method was needed, which could handle the highly similar gene content in the ResFinder databases. Initially, clustering of the database was considered with a workflow similar to that of SRST2 [33], as well as a sole mapping approach such as KmerResistance [34]. Both came with their own challenges, which gave rise to the development of KMA utilizing the novel ConClave algorithm to resolve highly similar sequences within a database [35]. In contrast to other alignment methods, KMA was developed from scratch with the task of aligning raw sequence data directly to redundant databases such as ResFinder. By applying an additional mapping step that identifies template candidates, the computational requirements were lowered with the bottleneck of AMR analysis from raw sequence data being the unzipping of input data. Adding KMA to the ResFinder pipeline reduced the analysis time of a usual WGS sample to <10 s, which in turn extended the lifespan of our web-servers now that *de novo* assembly can be avoided. Of course uploading raw sequence data to the web-tools from middle- or low-income countries remains a challenge.

### Predicting phenotypes

A large step was taken with version 4.0 of ResFinder [23], with not only identifying the different ARGs but also providing a prediction of the expected phenotypes for a selection of bacterial species. The foundation for this new feature was a new phenotype database with the interpretation of the phenotype for 3124 different gene variants that were obtained from the published literature or manually included based on similarity to gene variants with known phenotypes. Species-specific predictions for selected antimicrobial agents were also implemented in order to only present clinically relevant phenotypes for these species and also to include mutation-mediated phenotypes. Currently these include *Campylobacter coli, Campylobacter jejuni, Enterococcus faecalis, Enterococcus faecium, Escherichia coli, Mycobacterium tuberculosis, Salmonella* and *Staphylococcus aureus *. For these species, a species-relevant prediction is given but it is also possible to see all predictions. For species not mentioned here, all predictions are presented. In addition to adding the phenotype feature, the PointFinder database was also updated along with the ResFinder application, to hold and process information on situations where different combinations of multiple mutations or presence of resistance genes in combinations with specific mutations are needed to cause resistance.

The benchmarking that was done for the publication of ResFinder 4.0 showed concordance between the genotypical predicted and phenotypically detected phenotypes above 95% on overall average. In individual species-antimicrobial combinations concordance were for a few combinations below 95%. Most of these discrepancies were believed to be related to issues with the phenotypic cut-off values and/or the read depth of the NGS data. In the publication it was argued that the WGS approach is just as reliable as phenotypic testing, at least for surveillance.

### Use and curation of ResFinder

**Fig. 1.**
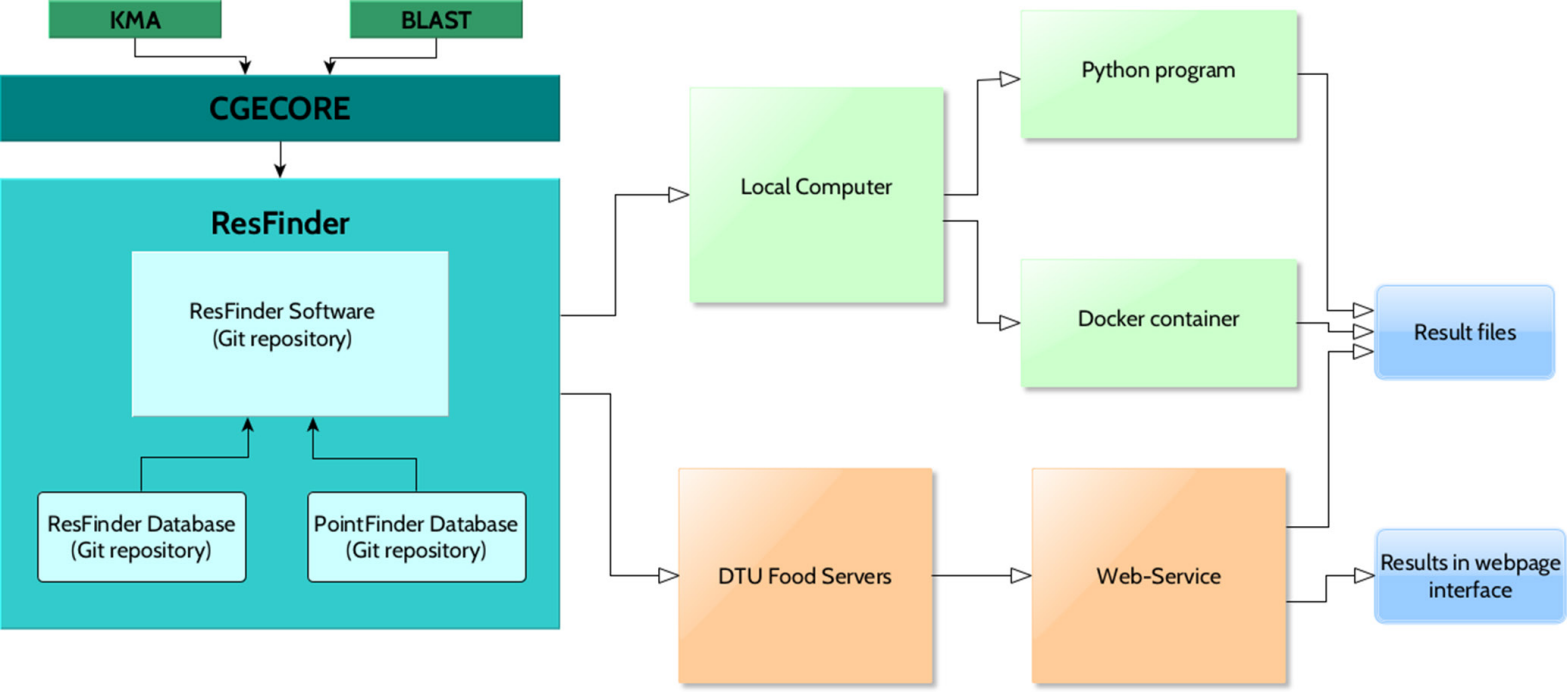
Architecture of ResFinder and the platforms available for users of the application.

The current version of ResFinder is built on three different Git repositories, separating the databases of acquired genes (the ResFinder database), the point mutations (the PointFinder database) and the ResFinder application (the ResFinder software) (Fig. 1). This allows users with bioinformatic experience to run the service offline and locally on their computers, as well as being able to incorporate it in their own pipelines as desired. In order to facilitate portability, the repository contains a DockerFile too.

However, to fulfil the original objective of ResFinder it is also provided for users with limited bioinformatics abilities through the web-based tool among the other CGE-tools. The web-page provides an interactive and easy interface, and does not require any programming knowledge, without missing any functionality of ResFinder. The web-service is hosted physically at DTU and the operating system is maintained by part-time system administrators to ensure that the web-service is as stable as possible.

In order to solve the issues and answer questions of the users, a database curator and a small team of part-time bioinformaticians (technical team) are ready to assist through the email given on the web-page and also respond to issues raised on Bitbucket.

### ResFinder software

**Fig. 2.**
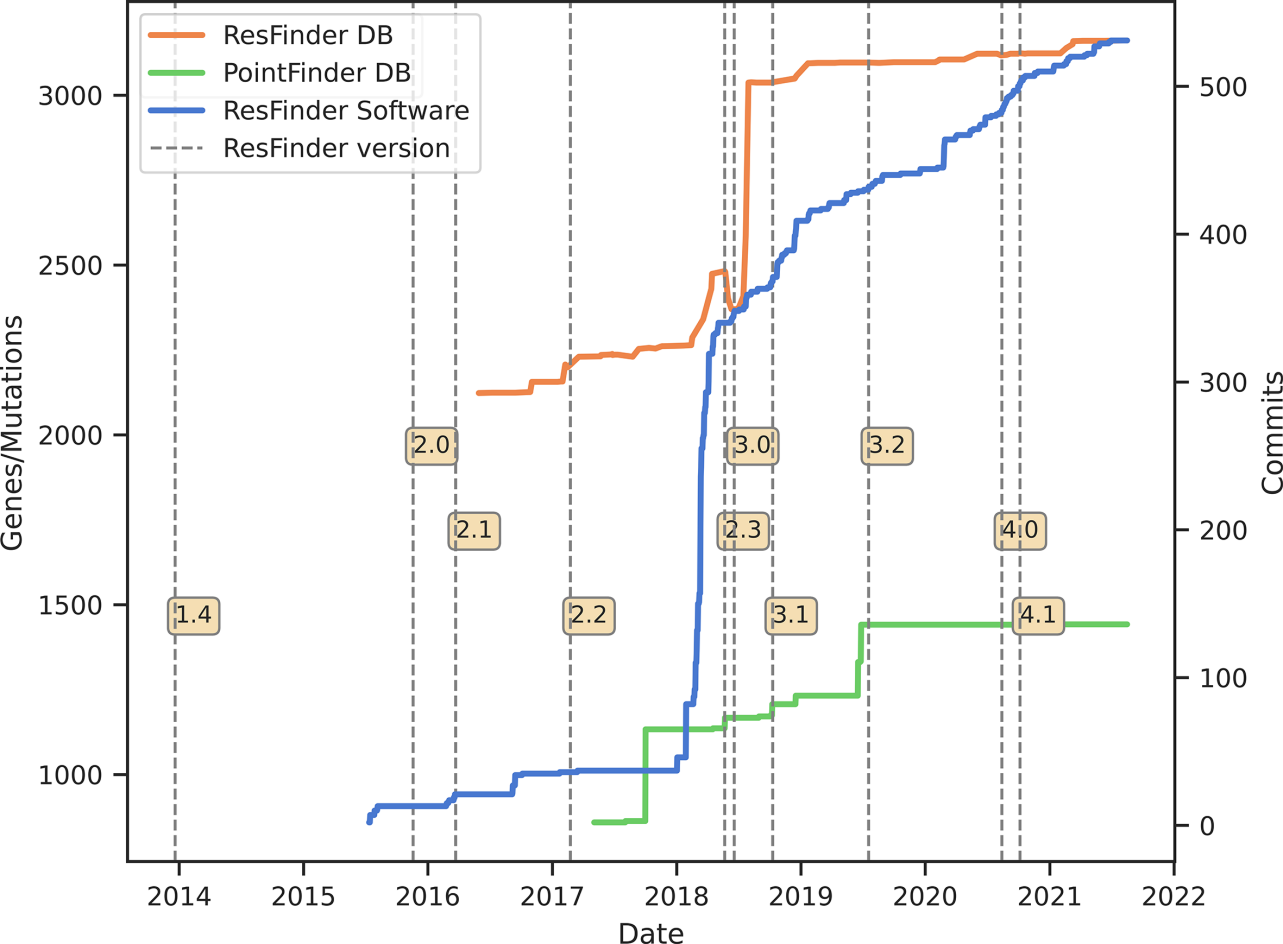
The version history and changes to the tool, as well as the databases. The blue line shows the number of updates (commits) in the software, the orange line, the number of genes in the ResFinder database and the green line the number of point mutations conferring antimicrobial resistance in PointFinder. The vertical dashed lines show the different versions of the ResFinder software.

The Python code behind ResFinder is built upon the CGECore package, which runs and integrates the results of the aligners KMA [35] and blast [29] for all the CGE-tools. ResFinder, the CGECore package and KMA are maintained and improved by a team of bioinformaticians and software developers. Fig. 2 shows the number of updates of the software and databases for the different versions.

As the ResFinder and PointFinder databases grow and become more complex, the software is constantly undergoing a process of updating, to fit the particularities of the data and facilitate interpretation of the results. Moreover, the bioinformatics team works on including the new features and improvements in ResFinder, hopefully expanding its functionality. This includes proposals for new features of KMA to fulfil interesting requirements of the scientific team or users of ResFinder. This includes extended output options and features, allowing for more streamlined analysis of metagenomic samples. Currently, new options are being added to KMA for better handling of third-generation sequencing data, as more and more users start to utilize these sequencing technologies.

### ResFinder and PointFinder database

The ResFinder database contains the ARGs per antimicrobial class in FASTA format. Moreover, the database contains the files describing intrinsic bacterial resistance (‘phenotype_panels.txt’) and detailed descriptions of the genes (‘phenotypes.txt’) such as the name of the gene, the NCBI accession number, the given resistance against the antimicrobial(s) and antimicrobial class(es), the related PMID of the article, the resistance mechanism produced by the gene, and if there is another gene in epistasis for establishing the resistance.

The PointFinder database includes the description of mutations/genetic variation that produce antimicrobial resistance in certain genes of the species *Campylobacter, Enterococcus faecalis, Enterococcus faecium, Escherichia coli, Helicobacter pylori, Klebsiella, Mycobacterium tuberculosis, Neisseria gonorrhoeae, Plasmodium falciparum, Salmonella* and *Staphylococcus aureus *. The database contains substitutions, deletions and insertions, which can be in coding regions, promoters or ribosomal RNA. The description of each genetic variation includes the PMID of the article where the resistance was found, the method of resistance provoked by the variation, and required mutations for the resistance.

Both databases are curated by a researcher with a biological background by reviewing published articles comprehensively to verify whether the novel resistance genes or mutations fulfil the requirements to be part of our databases. Afterwards, the bioinformaticians update the database with the new entries, and test technical aspects of the formats and attachment of the ResFinder software to the databases. Besides the literature review, the communication with users and researchers has been essential for updating and curating our databases.

### Future updates

The updates coming in the near future will focus on improving the ResFinder command line tool and implement a new output format that will become standard across all CGE-tools and facilitate easier implementation of CGE-tools in workflows (e.g. SnakeMake, Nextflow, Cromwell, etc.).

In addition, focusing on the use of raw reads, future ResFinder development points towards the detection of mixed populations on a sample and quantification of genes. While this naturally is essential for metagenomics, the quantification of mutations in multi-copy genes is also important to detect resistance, such as linezolid resistance in *Enterococcus* [36] and clarithromycin resistance in *Helicobacter pylori* [37].

We are continuously providing assistance in order to accommodate users with different backgrounds. Future versions of the software are focusing on allowing more flexibility in the parameters for detecting genes or point mutations, while providing mechanisms to not overwhelm the inexperienced users. Additionally, we are working on increasing the amount of platforms from which ResFinder is already available, like BioConda or Singularity, in order to facilitate its use and inclusion in the pipelines or workspaces. Providing a wide range of platforms and different levels of flexibility is particularly important when we expect our less experienced users to become more confident and skillful in bioinformatics as they use ResFinder.

Finally, as time and funding allows work, we will keep expanding the number of bacterial species to include into our databases for phenotypic predictions. Since we do not have sufficient resources to handle all bacterial species in the world, we do encourage researchers around the world to contact us if they have a species-specific database providing links between mutational genotypes and phenotypes.

### Evaluation of the usefulness of ResFinder

**Fig. 3.**
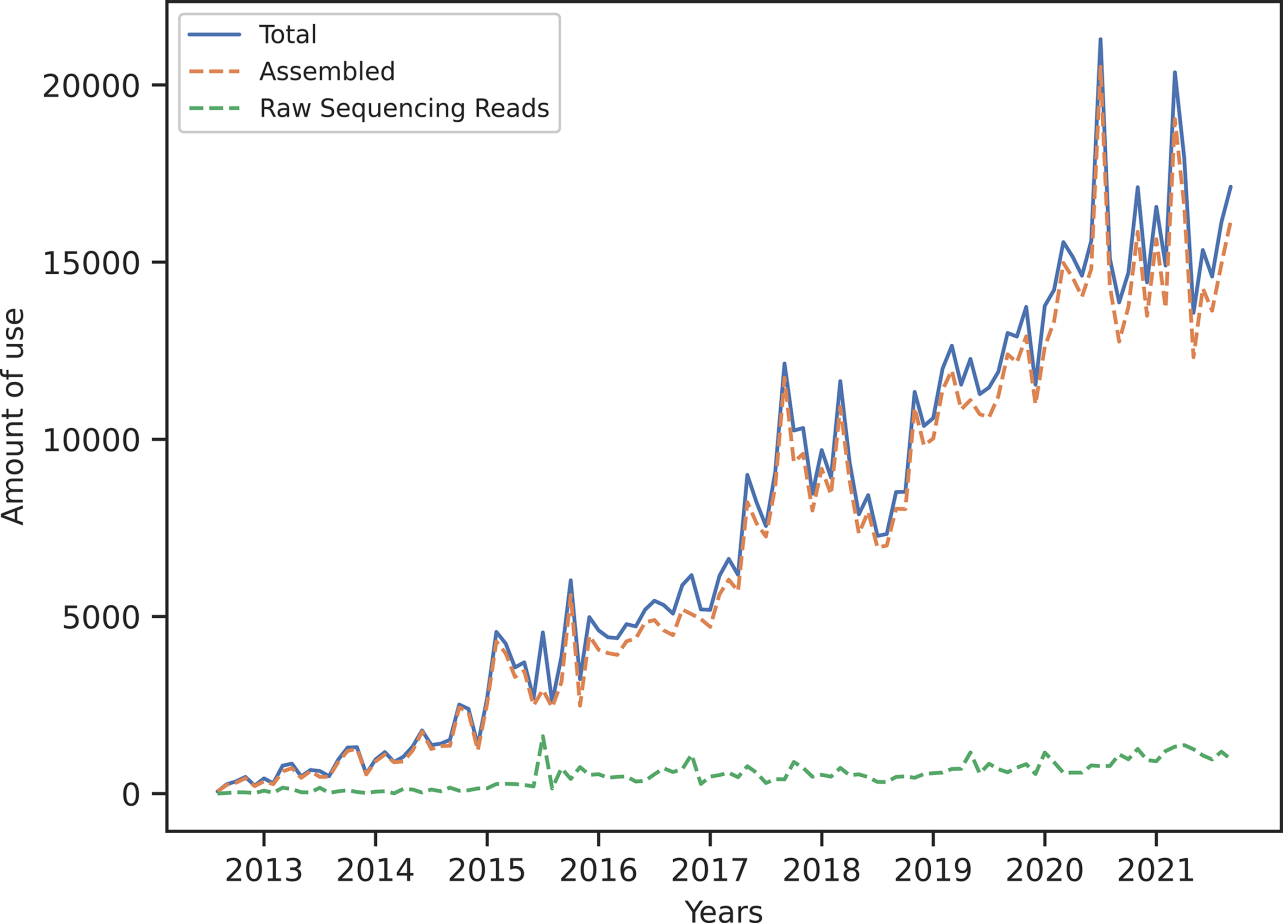
Monthly use of ResFinder over time. In the last 2 years, there have been large spikes in usage at the onset of the Covid pandemic. This can be explained by the transition of researchers (and less experienced users) to remote working/dry lab work because of mobility restrictions. We believe that this is evidence of ResFinder being accessible to the non-bioinformatician/less experienced user.

Number of scientific citations, downloads of databases and software might in many cases indicate the usefulness of different bioinformatic pipelines. However, since ResFinder was developed to promote the global use of NGS for research and diagnostic microbiology, especially in LMICs, the geographical use of the online tool is for us a better measure of usefulness. Since it was launched in 2012, the monthly usage of ResFinder has, with some fluctuations, increased steadily. Today, we process approximately 15 000 analyses per month of both raw and assembled sequences (Fig. 3). Most analyses are based on already assembled sequences and we expect this may be due to difficulties in uploading raw sequencing data over the internet. However, no further information is available for the purposes of analyses, as we do not store any of the user data.

**Fig. 4.**
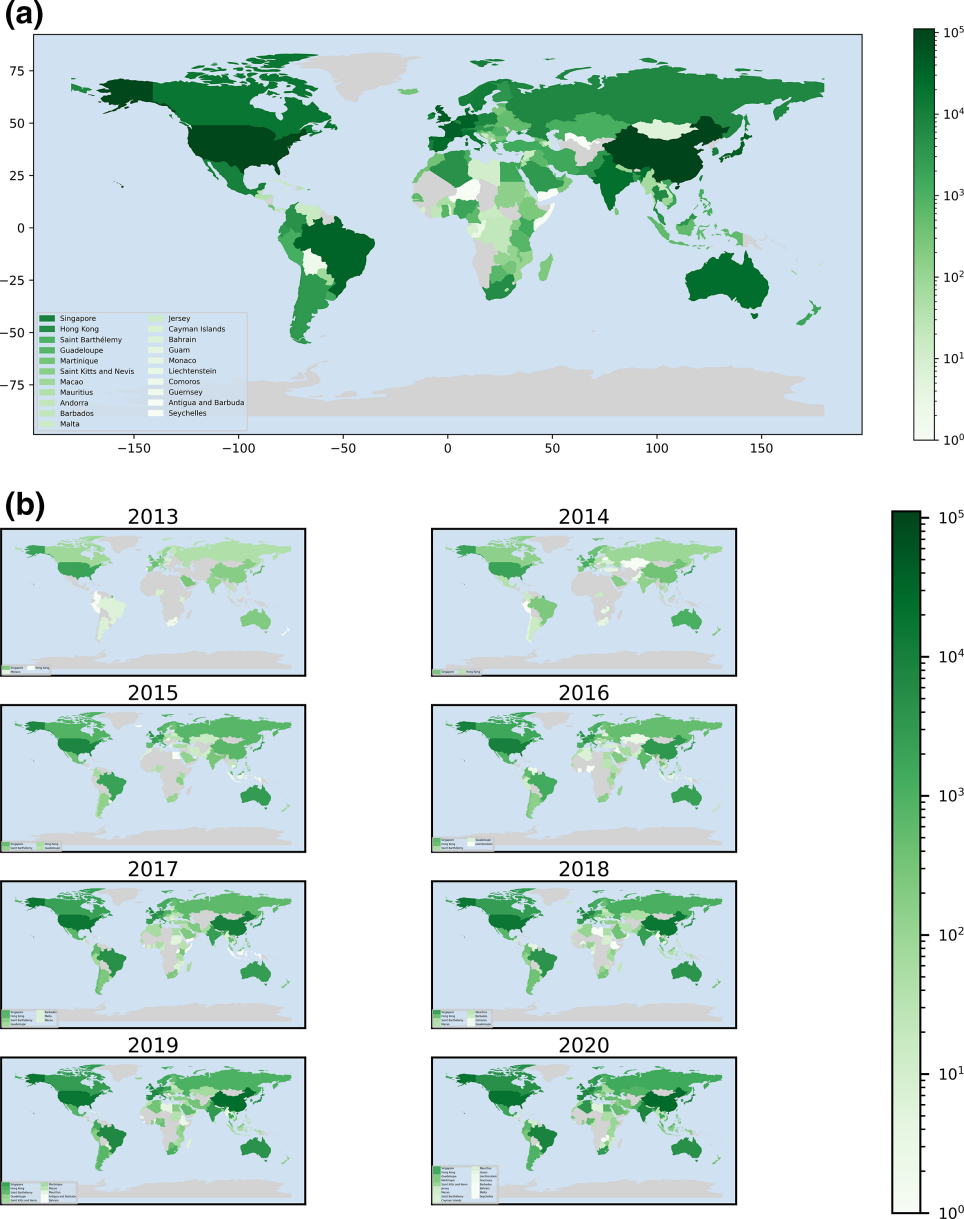
Geographical use of ResFinder. The cumulative use per country shown at the top and the use per year per country at the bottom.

The geographical use of ResFinder is shown in Fig. 4. To date (September 28th 2021) ResFinder has been used by 61 776 IP-addresses from at least 171 different countries to conduct 820 803 analyses. Particularly the USA, China and the countries in Western Europe have been extensively using ResFinder. Thus, it seems clear that even though ResFinder was developed with the aim to support researchers from LMICs, it has also been found useful by users from several other countries in the world.

**Fig. 5.**
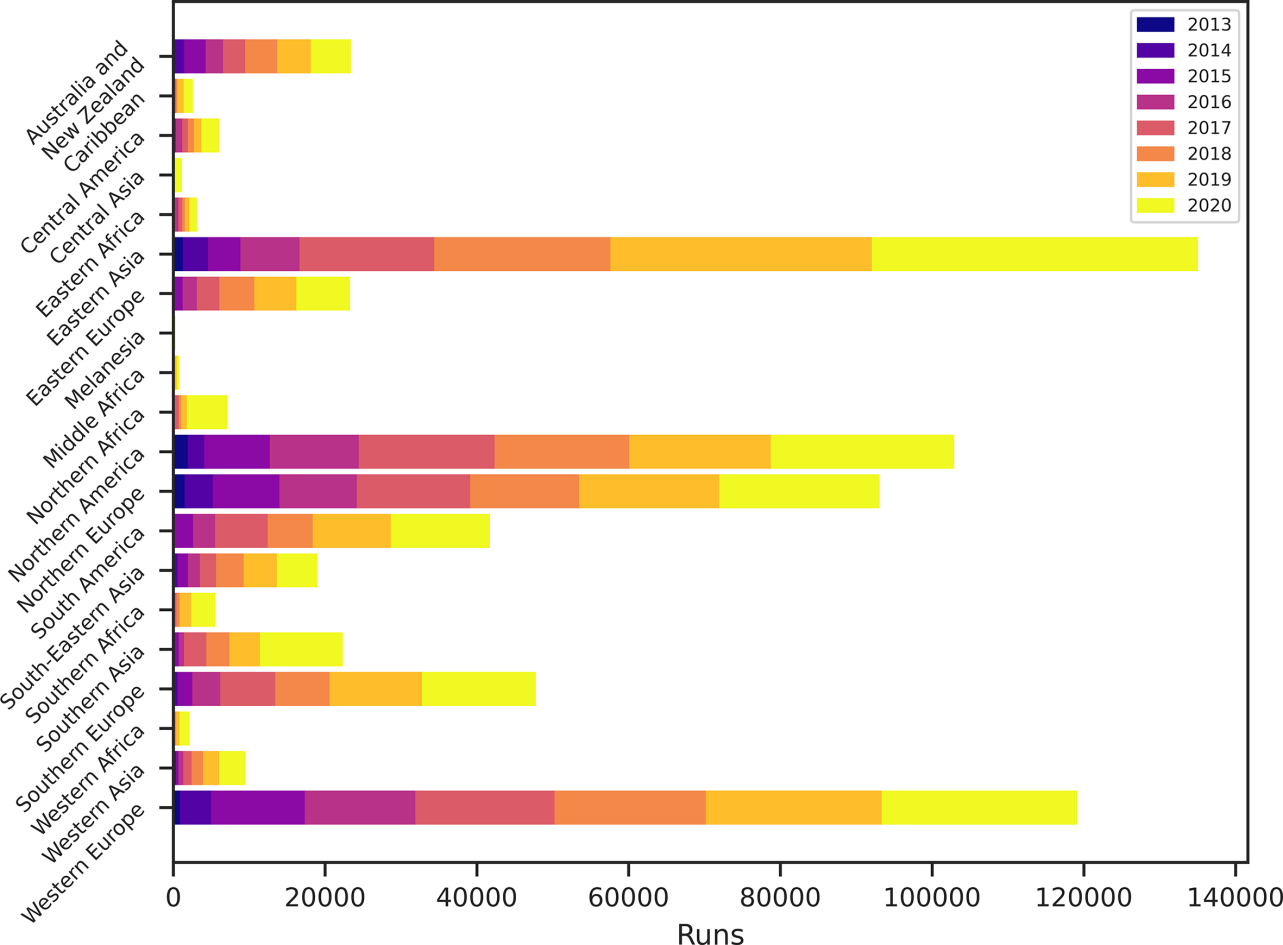
Cumulative use of ResFinder in different geographical regions over time.

The geographical use has also changed over time (Figs 4 and 5). Initially, a majority of the users were from North and Western Europe and North America. However, the predominant users have changed over time. Today, most of the users are from Asia, although other regions are having increased use. While users from LMICs are still a minority, they have started to become visible. The increased use from LMICs is encouraging for us and we hope to keep the facility running for open use for all, also in the future.

In addition to the use of the online service the Resinder database and software has also been downloaded by a number of users around the world. It is, however, very difficult to estimate the usefulness from this.

### From rule based methods to machine learning for predicting phenotypes

The manually curated databases of ResFinder and PointFinder provide antimicrobial resistance phenotypes for the selected species and antibiotic combinations by detecting the absence and presence of ARGs, and resistance conferring point mutations. The manual (rule-based) ResFinder and PointFinder tools provide highly accurate resistance phenotypes for the well studied organism and antimicrobial combinations. However, the accuracy of these tools are limited for the less studied combinations due to the insufficient knowledge regarding the resistance mechanisms [38]. Therefore, the success of the rule-based tools strictly depend on prior knowledge. Machine learning can potentially aid in solving this issue, by learning the resistance-bearing patterns from the phenotyped data itself. Machine learning is a group of automated pattern-detection methods that can be applied to biological problems to avoid or minimize human interference. To date, machine learning has been utilized successfully for prediction of antimicrobial resistance phenotypes, MICs and discovery of resistance genes [38–43]. Previous studies proved that machine learning can be used as an alternative way to detect antimicrobial resistance by avoiding the sensitive and expensive empirical studies for detecting resistance conferring genes and mutations, while enabling the detection of epistatic interactions. Nevertheless, machine learning comes with its own caveats, such as data quality and high computational requirements. It is not completely independent of prior knowledge. Known resistance phenotypes are required for the supervised machine-learning models. With the exponentially growing data, the data-driven approaches such as machine learning have started to replace the manual rule-based approaches. ResFinder has also started to work on integrating the machine-learning-based antimicrobial resistance phenotype prediction models to enhance the tool accuracy and increase the availability of the number of organisms and antibiotic combinations. This integration requires careful interpretation of the probabilistic machine-learning results and simultaneously comparison to the rule-based approaches.

### Future and conclusion

A number of different ARG-databases exist as do a number of different analytic pipelines for using the databases and analysing NGS data [9]. It may seem redundant to have so many different solutions and that work towards global harmonization could be appropriate. However, it should also be noted that currently the different solutions do fulfil different needs and niches and are not directly competing, but to a large extent complementary. More collaboration would however, be appropriate also to limit the resources spent on providing these solutions.

In the future we expect to expand the number of bacterial species where we provide clinical predictions, integrate ResFinder into a pipeline and provide batch upload solutions and to move from rule-based to machine-learning approaches. As long as the global need is there and especially as long as our services can help building capacity in LMICs we hope to keep the services updated, relevant and running.
